# Real‐world evidence of brigatinib as second‐line treatment after crizotinib for *ALK*+ non‐small cell lung cancer using South Korean claims data (K‐AREAL)

**DOI:** 10.1002/cam4.70030

**Published:** 2024-07-18

**Authors:** Jeong Eun Lee, Jin Hyun Nam, Sun Hong Kwon, Bo Kyung Kim, Seung Min Ha

**Affiliations:** ^1^ Division of Pulmonology, Department of Internal Medicine, College of Medicine Chungnam National University Daejeon Korea; ^2^ Division of Big Data Science Korea University Sejong Korea; ^3^ School of Pharmacy Sungkyunkwan University Suwon Korea; ^4^ Takeda Pharmaceuticals Korea Co., Ltd. Seoul Korea

**Keywords:** brigatinib, claims data, effectiveness, real‐world evidence, safety, South Korea

## Abstract

**Purpose:**

There is a lack of real‐world data in Asian populations for brigatinib, a next‐generation anaplastic lymphoma kinase (ALK) inhibitor for patients with non‐small cell lung cancer (NSCLC). This study analysed real‐world outcomes and dosing patterns for brigatinib in patients with crizotinib‐refractory *ALK*+ NSCLC in South Korea.

**Methods:**

This retrospective, non‐interventional, cohort study used South Korean Health Insurance and Review Assessment claims data for adults with *ALK+* NSCLC who initiated brigatinib between 19 April 2019 and 31 March 2021 after receiving prior crizotinib. Patients' characteristics, time to discontinuation (TTD), time to dose reduction, overall survival (OS) and treatment adherence were assessed.

**Results:**

The study included 174 patients (56.9% male; 27.0% with a history of brain metastases). Median duration of prior crizotinib was 17 (range 0.3–48) months. Median follow‐up after brigatinib initiation was 18 (range 0–34) months. Overall, 88.5% of patients received full‐dose brigatinib (180 mg/day) and 93.1% of patients were adherent (proportion of days covered ≥0.8). The median TTD was 24.9 months (95% CI 15.2–not reached). The probability of continuing treatment was 63.2% at 1 year and 51.5% at 2 years. The probability of continuing at full or peak dose was 79.7% at 1 year and 75.6% at 2 years. Median OS was not reached. The 2‐year OS rate was 68.7%.

**Conclusions:**

In this first nationwide retrospective study using national insurance claim data, brigatinib demonstrated real‐world clinical benefit as second‐line treatment after prior crizotinib in *ALK*+ NSCLC patients in South Korea.

## 1 INTRODUCTION

Lung cancer is one of the most common cancers worldwide, and the leading cause of cancer‐related death.^1^ In South Korea, the age‐standardised incidence rate for lung cancer was 25.8 per 100,000 population in 2020 and it caused more than 18,000 deaths per year.^2^

Non‐small cell lung cancer (NSCLC) accounts for 85% of lung cancer cases.^3^ Patients in whom driver mutations are found may be suitable for targeted therapy. Anaplastic lymphoma kinase (ALK) rearrangements, which are found in 3%–5% of NSCLC cases,^4^, ^5^ are associated with aggressive histology, younger age, limited or no history of smoking, and a high risk of brain metastases.^6^ Over the last decade, the therapeutic landscape for *ALK*‐positive (*ALK*+) NSCLC has witnessed a paradigm shift, with the advent of ALK‐tyrosine kinase inhibitors (ALK‐TKIs).^7^, ^8^

Crizotinib, the first ALK inhibitor, was followed by second‐generation ALK inhibitors, which were developed to overcome crizotinib resistance and improve efficacy, particularly in the CNS.^8^ Brigatinib is a potent and selective second‐generation ALK inhibitor.^9^ In a phase 2 trial (ALTA), brigatinib exhibited clinical efficacy in patients with *ALK+* NSCLC refractory to crizotinib, with an independent review committee (IRC)‐assessed objective response rate (ORR) of 56%, intracranial ORR of 67%, and median progression‐free survival (PFS) of 16.7 months for patients treated with a dose of 180 mg (after a 7‐day 90 mg lead‐in).^10^ In a phase 3 trial (ALTA‐3), median IRC‐assessed PFS was 19.3 months, the ORR was 52%, and the intracranial ORR was 73% with brigatinib given as second‐line treatment to patients with *ALK+* NSCLC who had progressed on crizotinib.^11^ Brigatinib has also demonstrated efficacy in ALK inhibitor‐naïve patients, with a significantly longer IRC‐assessed median PFS compared with crizotinib (24.0 vs. 11.1 months; HR 0.48, *p* < 0.0001) in a phase 3 trial (ALTA‐1L).^12^

Currently, only limited real‐world evidence for brigatinib has been reported. Available data from the USA, Europe and South America indicate that brigatinib is effective and well tolerated in routine clinical practice.^13^, ^14^, ^15^, ^16^, ^17^ However, as well as including data for second‐line brigatinib, most current real‐world studies include later lines of brigatinib. In addition, there is a lack of real‐world data in Asian populations. Brigatinib was approved in South Korea in November 2018 (and received reimbursement on 1 April 2019) for the second‐line treatment of patients with locally advanced or metastatic *ALK+* NSCLC who have progressed on crizotinib. First‐line brigatinib was approved in August 2020 and it has been reimbursed since 1 April 2021. The aim of the current study (K‐AREAL) was to analyse real‐world dosing patterns and treatment outcomes for brigatinib in patients with crizotinib‐refractory *ALK*+ NSCLC in South Korea.

## 2 METHODS

This retrospective, non‐interventional cohort study analysed claims data from Health Insurance and Review Assessment (HIRA), the national health insurance database that covers almost the entire population of South Korea. The study was approved by the institutional review board of Sungkyunkwan University, Suwon, Republic of Korea. Informed consent was not required because the study used anonymised and de‐identified claims data.

### 2.1 Study population

**FIGURE 1:**
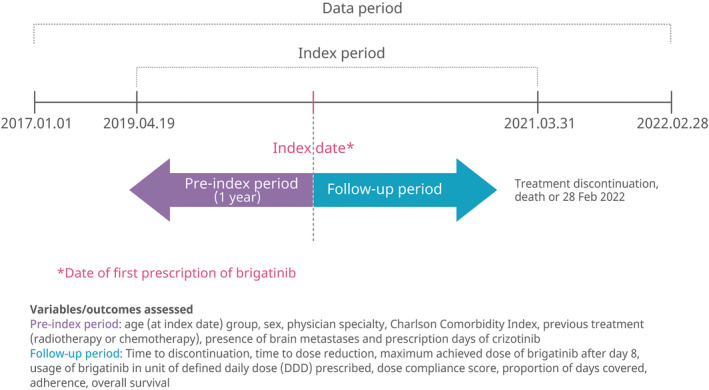
Study design.

Figure 1 provides an overview of the study design. The study included adults (aged ≥20 years) with *ALK+* NSCLC who started treatment with brigatinib between 19 April 2019 and 31 March 2021 as second‐line therapy after receiving crizotinib. Patients had to have had at least two claims with a diagnosis of lung cancer (International Classification of Diseases, ICD‐10 code C34) during the study period. Patients who had received a prior ALK inhibitor other than crizotinib before the initiation of brigatinib were excluded. Patients were followed up from the date of first prescription of brigatinib (index date) until time to treatment discontinuation, death, or 28 February 2022. The 1‐year period preceding the index date was defined as the pre‐index period. The approved recommended dosing regimen for oral brigatinib is 180 mg once daily with a 7‐day lead‐in at 90 mg once daily and dose reduction to 120, 90 and 60 mg, according to the severity of adverse events.

### 2.2 Variables

The following outcomes were evaluated. Patient characteristics, including age, sex, Charlson Comorbidity Index (based on claims during the pre‐index period), previous treatment (radiotherapy or chemotherapy during the pre‐index period) and the presence/absence of brain metastases (during the pre‐index period; defined by the diagnosis code C793 [secondary malignant neoplasm of brain and cerebral meninges]). Time to discontinuation (TTD; primary endpoint), defined as the number of days between initiation and discontinuation of brigatinib (with discontinuation identified by a gap of ≥90 days in brigatinib therapy or initiation of another ALK TKI). Time to dose reduction (TTDR), defined as the number of days from brigatinib initiation to dose reduction (with dose reduction defined as the first claim for a prescription of a 30 or 90 mg tablet after prescription of 180 mg or, for patients who did not reach a dose of ≥180 mg/day, the first claim for a prescription of a daily dose less than the maximum achieved dose). The probability of patients continuing brigatinib and the probability of continuing brigatinib at full dose (or peak dose if receiving <180 mg) were estimated at 6, 12 and 24 months. To assess overall survival (OS), death was defined via death‐related ICD‐10 diagnosis codes or when there were no claims within 1 year of the last claim.^18^ Adherence to brigatinib treatment in real‐world practice was also evaluated, including the proportion of days covered (PDC; calculated as the proportion of days in which a person had access to medication over the period of interest) and adherence (patients with a PDC of ≥0.8 were defined as treatment adherent).

Post hoc subgroup analyses were performed based on the presence/absence of brain metastases and whether patients continued brigatinib for 4 months after the index date (with or without a dose reduction). The latter analysis was conducted to better assess the efficacy of the drug due to the limitations of a real‐world study, in which it is difficult to distinguish the reason for discontinuation of brigatinib: based on clinicians' experience and published literature, patients treated with targeted anticancer drugs such as TKIs generally develop adverse events within the first 3–4 months of drug initiation^19^, ^20^, ^21^, ^22^, ^23^, ^24^, ^25^, ^26^; therefore, during that period there may be a greater chance that patients will discontinue treatment due to tolerability, whereas beyond that timepoint there may be a greater chance that patients will discontinue due to disease progression rather than due to tolerability.

### 2.3 Statistical analysis

Data were summarised using descriptive statistics, including mean, standard deviation (SD), median, minimum, and maximum for data with normal distribution, and frequency and percentage for categorical variables. Kaplan–Meier methodology was used to estimate time‐to‐event outcomes, including TTD, TTDR and OS. The probability of continuation on brigatinib and continuation on a maximum dose of brigatinib at 6, 12 and 24 months were evaluated using Kaplan–Meier analyses. Statistical analyses were performed using SAS® version 7.1 and R version 3.5.1.

## 3 RESULTS

**FIGURE 2:**
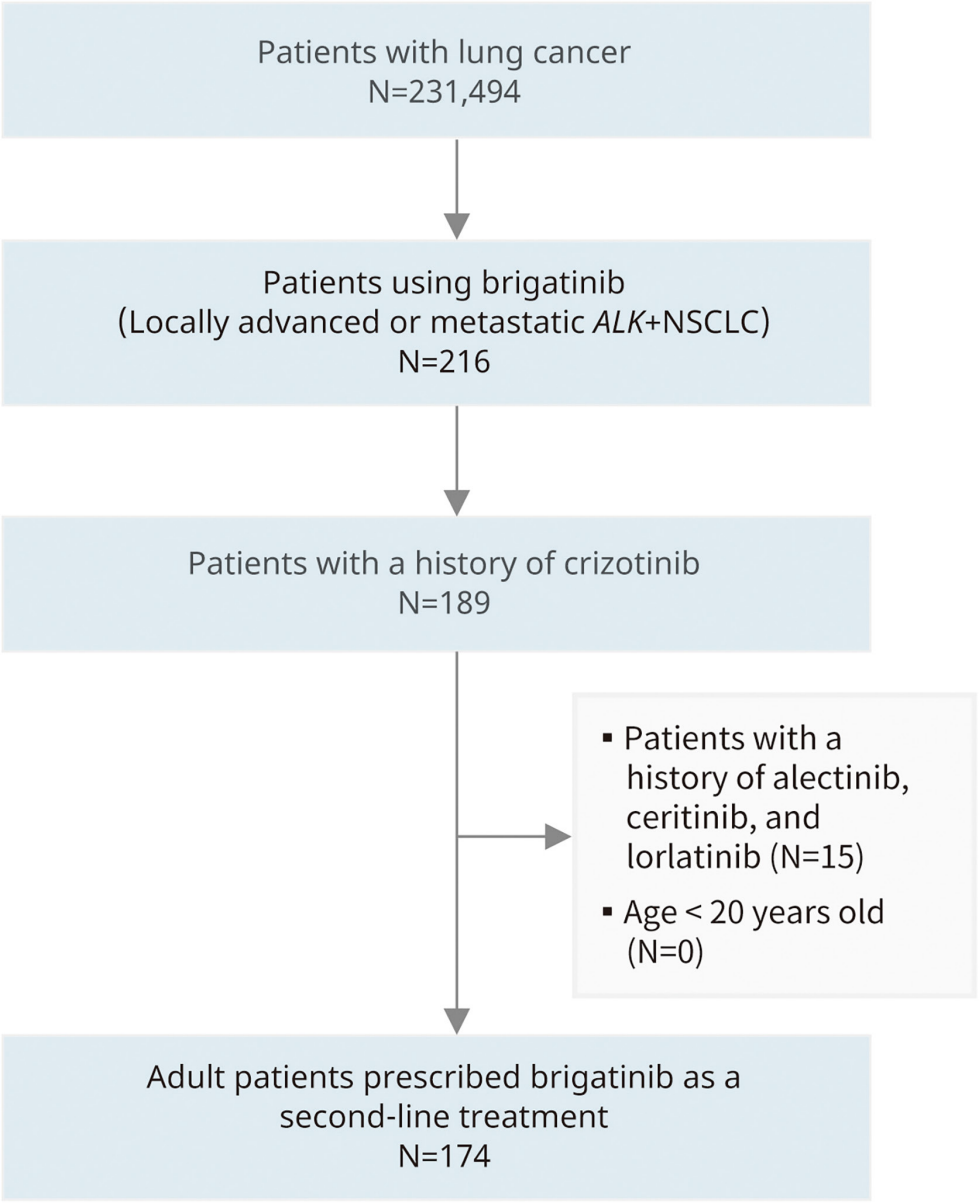
Patient selection process.

A total of 174 crizotinib‐refractory patients with *ALK*+ NSCLC who received brigatinib as second‐line therapy were included in the analysis (Figure 2).

Almost half of the patients (46.6%) were aged <60 years, 56.9% were male, the median Charlson Comorbidity Index score was 4.00 (range 0.00–11.00), and 27.0% had a history of brain metastases in the pre‐index period. The median duration of prior crizotinib treatment was 17 (range 0.3–48) months, and the median time between crizotinib and brigatinib use was 2.1 (range 0.06–35) months. Fourteen patients (8.0%) had a gap of >180 days between the last claim for crizotinib and the first claim for brigatinib.

The median duration of follow‐up in the study was 18 (range 0–34) months. Overall, 88.5% of patients reached the maximum dose of brigatinib 180 mg. The median PDC was 0.98 (range 0–1.00), and 93.1% of patients were classified as adherent to brigatinib (Table 1).

**TABLE 1:** Patient characteristics.

Variable^a^	Number of patients (%^b^), *N* = 174
Age, years (index date)	
<50	29 (16.7)
50–59	52 (29.9)
60–69	47 (27.0)
70–79	38 (21.8)
80+	8 (4.6)
Sex	
Female	75 (43.1)
Male	99 (56.9)
Charlson Comorbidity Index	
0	32 (18.4)
1	22 (12.6)
2	15 (8.6)
3	17 (9.8)
4+	88 (50.6)
Median (min–max)	4.00 (0.00–11.00)
Brain metastases^c^ (pre‐index), yes	47 (27.0)
Prior chemotherapy (pre‐index), yes	6 (3.5)
Prior radiation (pre‐index), yes	19 (10.9)
Duration of crizotinib use, months^d^	
Median (min–max)	17.0 (0.3–48)
Gap between crizotinib and brigatinib^e^, yes	14 (8.0)
Duration of follow‐up, months	
Median (min–max)	18 (0–34)
Brigatinib maximum dose achieved (mg)	
180	154 (88.5)
120	1 (0.6)
90	19 (10.9)
60	0 (0)
Proportion of days covered^f^ (brigatinib)	
Median (min–max)	0.98 (0–1.00)
Adherence to brigatinib	
PDC ≥0.8	162 (93.1)
PDC <0.8	12 (6.9)

Abbreviations: PDC, proportion of days covered; SD, standard deviation.

^a^ Assessed at index date or during pre‐index period (1‐year period preceding index date).

^b^ Data are % unless indicated otherwise.

^c^ Defined by diagnosis code C793 (secondary malignant neoplasm of brain and cerebral meninges).

^d^ Days using crizotinib between 1 January 2017 and index date.

^e^ Gap defined as >180 days between the last claim for crizotinib and the first claim for brigatinib.

^f^ Proportion of days in which a person had access to medication over the period of interest.

**FIGURE 3:**
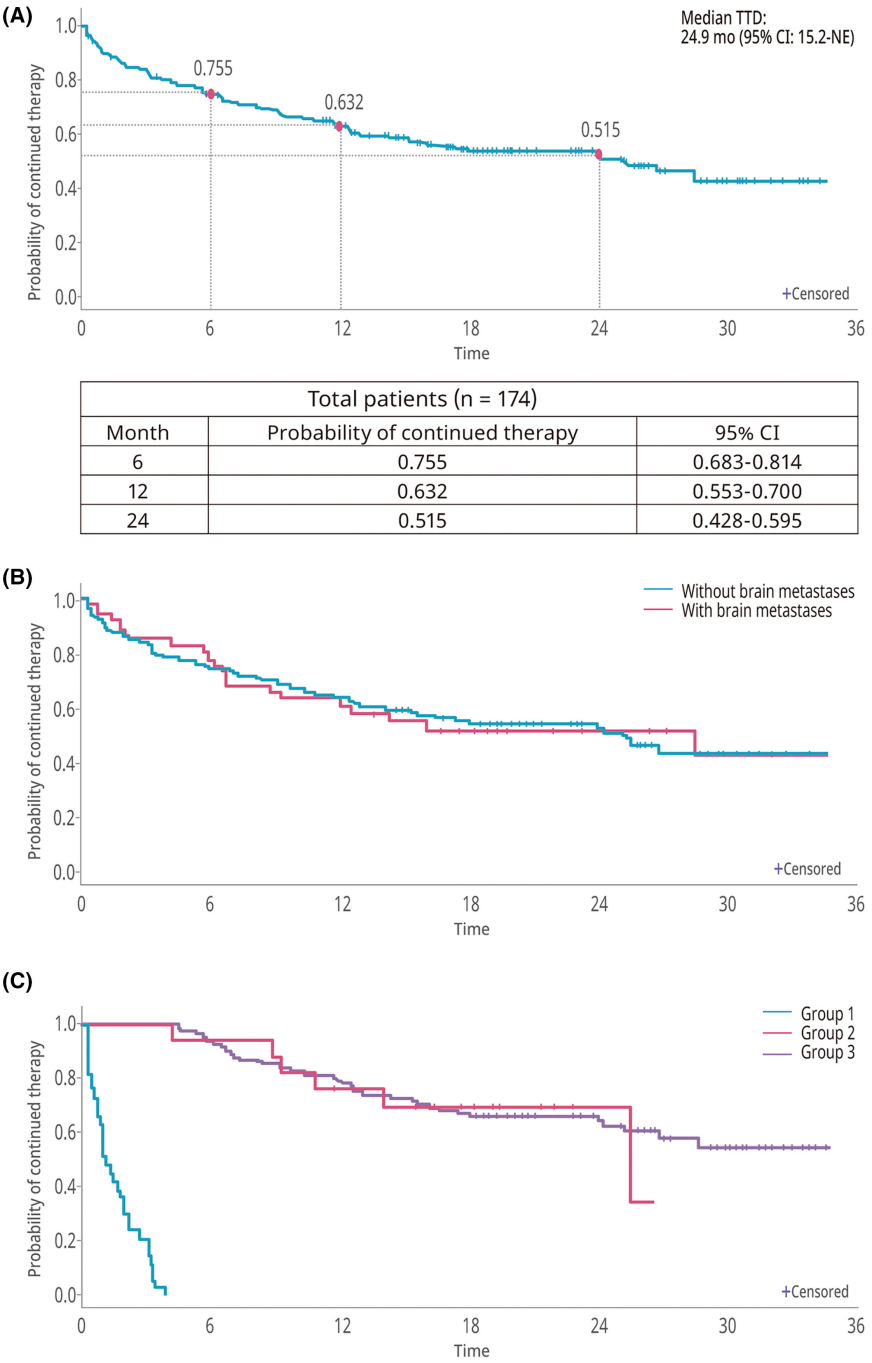
Time to discontinuation of brigatinib. (A) Total study population. (B) With or without brain metastases. (C) According to brigatinib discontinuation status (Group 1: discontinued within 4 months [*n* = 33]; Group 2: continued to 4 months with dose reduction [*n* = 18]; Group 3: continued to 4 months without dose reduction [*n* = 123]).

The median TTD was 24.9 months (95% CI 15.2–not reached [NR]), and the probability of continuing treatment at 1 and 2 years was 63.2% and 51.5%, respectively (Figure 3A). TTD did not differ significantly between patients with and without brain metastases (median [95% CI] = 28.3 months [9.2–NR] vs. 25.1 months [15.1–NR]; *p* = 0.983; Figure 3B). As would be expected, median TTD was significantly longer in patients who continued brigatinib for at least 4 months (patients without dose reduction [*n* = 123]: NR, 95% CI 26.6–NR; patients with dose reduction [*n* = 18]: 25.3 months, 95% CI 10.7–NR) compared with those who discontinued within 4 months (*n* = 33; 1.1 months, 95% CI 0.7–1.9) (*p* < 0.0001; Figure 3C).

**FIGURE 4:**
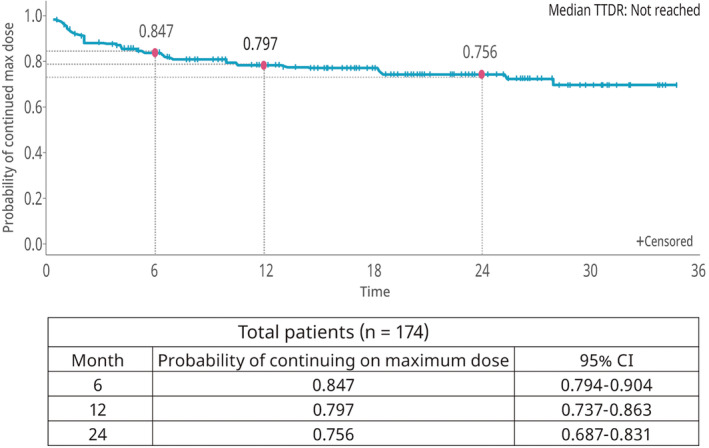
Time to brigatinib dose reduction (total study population).

The probability of continuing at the maximum dose (180 mg/day) or at the peak dose (if <180 mg/day) was 84.7% at 6 months, 79.7% at 1 year and 75.6% at 2 years (Figure 4).

**FIGURE 5:**
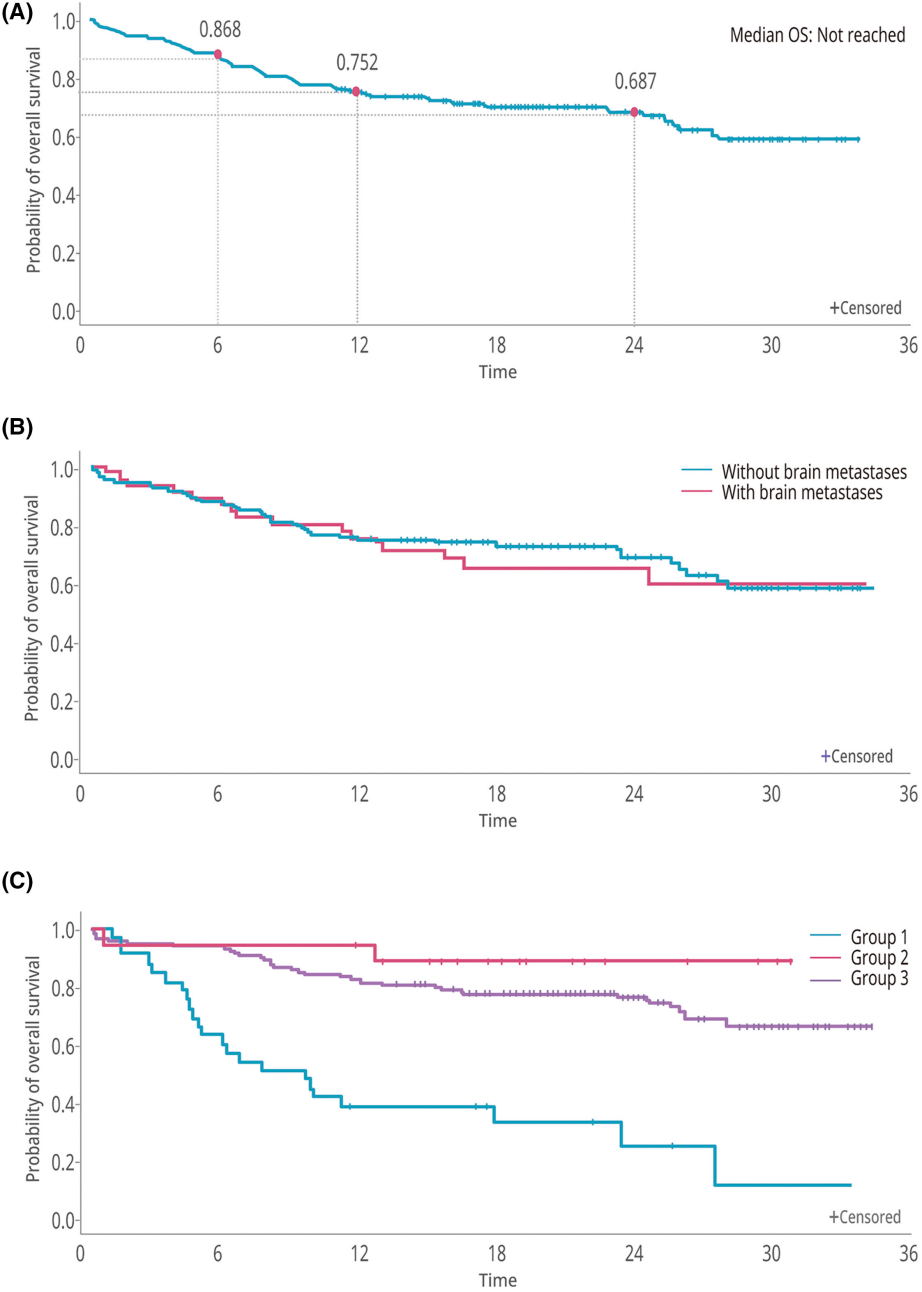
Overall survival. (A) Total study population. (B) With or without brain metastases. (C) According to brigatinib discontinuation status (Group 1: discontinued within 4 months [*n* = 33]; Group 2: continued to 4 months with dose reduction [*n* = 18]; Group 3: continued to 4 months without dose reduction [*n* = 123]).

Median OS was not reached (95% CI 27.6 months–NR) during the study period (Figure 5A). The 2‐year OS rate was 68.7%. The survival benefit of brigatinib was similar in patients with or without brain metastases (*p* = 0.7796; Figure 5B), whereas median OS was lower in patients who discontinued brigatinib within 4 months compared with patients who continued the drug for 4 months, even if they received a reduced dose (9.4 months vs. NR; *p* < 0.0001; Figure 5C).

## 4 DISCUSSION

This study represents the first real‐world evidence on the use of brigatinib in crizotinib‐refractory patients with *ALK*+ NSCLC in South Korea. The study used nationwide national claims data from HIRA, which covers fee‐for‐service payments for almost all citizens in the country; therefore, the analysis included a large cohort of 174 patients and had a relatively long median follow‐up period of 18 months. The strict reimbursement criteria imposed by HIRA meant that the study population was well‐defined, comprising patients who moved from first‐line crizotinib to second‐line brigatinib.

With respect to patient characteristics, the proportion with a history of brain metastases in the current study (27%) was lower than that reported for brigatinib recipients in a phase 3 trial (ALTA‐3) in crizotinib‐refractory patients (64%)^11^ but similar to that in the pivotal phase 3 trial (ALTA‐1L) of brigatinib in ALK inhibitor‐naïve patients (29%).^27^ Differences may reflect the fact that patients with brain metastases could have been missed if those characteristics were not detected in the claims data. In the current study, 3.5% of patients had received prior chemotherapy compared with 31% in ALTA‐3 and 27% in ALTA‐1L.^11^, ^27^ This may be due to the change in treatment paradigm for *ALK+* NSCLC since these trials commenced, with a move away from chemotherapy and towards targeted therapies. The median duration of prior treatment with crizotinib in the current study (17.0 months) was longer than the IRC‐assessed median PFS of crizotinib as first‐line treatment in other trials: 7.7 months (PROFILE 1007 trial),^28^ 10.4 months (ALEX trial)^29^ and 11.1 months (ALTA‐1L trial),^12^ but consistent with the 16 months reported for the ALTA‐3 trial.^11^

The median TTD of 24.9 months for brigatinib in the current study was longer than the duration of brigatinib treatment in other real‐world studies and PFS in clinical trials. In a multinational expanded access programme, the brigatinib TTD after one prior line of ALK inhibitor was 11.8 months,^16^ while in US and Latin American real‐world studies the TTD of brigatinib given as second or later line of therapy was 10.3 months^13^ and 18.5 months,^15^ respectively. TTD is a pragmatic endpoint used to assess treatment duration in real‐world studies that correlates with PFS in clinical trials of metastatic NSCLC.^30^ TTD (median 24.9 months) in the current study was longer than PFS reported in clinical trials of second‐line brigatinib in crizotinib‐refractory patients; median IRC‐assessed PFS with brigatinib 180 mg/day was 16.7 months in the final analysis of ALTA^10^ and 19.3 months in ALTA‐3.^11^ Several factors can be postulated to explain these differences in treatment duration, including study design and patient population differences between real‐world and clinical trial settings, as well as differences in the national health insurance system compared with other countries and proactive treatment attitudes in South Korea.

Median OS was not reached during the evaluation period for the current study, but the 2‐year OS rate of 68.7% suggests that brigatinib could provide extended survival in many patients when used as second‐line ALK inhibitor therapy. In the ALTA trial, median OS (IRC‐assessed) was 40.6 months and the probability of survival at 5 years was 43% for patients treated with brigatinib 180 mg,^10^ while in ALTA‐3, median OS was not reached and the 1‐year OS rate was 89% in the brigatinib arm.^11^ The current study included a subgroup analysis based on brigatinib continuation status at 4 months. Median OS was lower in the subgroup of patients who discontinued brigatinib within 4 months compared with those who continued brigatinib for at least 4 months. This suggests that it may be advisable to maintain brigatinib treatment, if possible, to improve OS. Furthermore, TTD did not differ between patients with or without a dose reduction within 4 months, suggesting that similar clinical benefit in terms of delaying disease progression could be achieved even with a reduced dose. Data from a real‐world study (RESET) of afatinib in patients with NSCLC reported a similar extension of treatment duration and OS through dose adjustment. The study showed that dose adjustment in most patients (62.3%) due to side effects resulted in better treatment outcomes.^31^ Therefore, if patients experience adverse events during the first 3–4 months, proactive consideration should be given to a dose reduction to enable treatment to be continued. However, it should be noted that, in the current study, there was only a small number of patients in the subgroup who continued brigatinib to 4 months with a dose reduction, limiting the interpretation of this finding. Moreover, although the current study showed a similar TTD in the dose reduction group, the approved recommended dosing regimen for oral brigatinib, if possible, is 180 mg once daily with a 7‐day lead‐in at 90 mg once daily.^32^ Rather than stopping brigatinib due to side effects, it is recommended to actively consider a strategy to reduce the dose.

Patients with *ALK*+ NSCLC have a high risk of brain metastases at the time of diagnosis of advanced disease or within a few years.^33^ Second‐generation ALK inhibitors are more effective than crizotinib in patients with brain metastases.^34^, ^35^ Typically, patients with brain metastases will have a shorter treatment duration; however, in the current study, TTD was similar whether or not patients had brain metastases. This is consistent with findings from the phase 2 ALTA clinical trial in crizotinib‐refractory patients, in which no notable difference in PFS was found between patients with or without brain metastases at baseline.^10^ In the current study, the survival benefit did not differ significantly between patients with or without brain metastases. Again, this is consistent with data from the ALTA trial, which showed numerically longer OS in patients who had brain metastases at baseline compared with those who did not.^10^ Taken together, these results suggest brigatinib shows good efficacy even in patients with brain metastases.

Adherence to ALK inhibitor therapy in a real‐world setting is generally good.^36^ Most patients in the current study (88.5%) reached the full dose of brigatinib (180 mg/day), and most patients (93.1%) were adherent to treatment. This is consistent with a US real‐world study (in which >80% had received a prior ALK inhibitor), where 77% of patients reached brigatinib 180 mg/day and 92.7% of patients were adherent.^13^ The probability of continuing brigatinib therapy at 1 year in the current study (63.2%) was higher than when it was administered as second‐line therapy in the global expanded access programme (49.3%) or as second or later line therapy in the US (45%) and Latin American (59.9%) real‐world studies.^13^, ^15^, ^16^ It is possible this could indicate a difference between Asian and non‐Asian populations, which would be consistent with the ALTA‐1 L clinical trial of brigatinib in ALK inhibitor‐naïve patients, which found that PFS was longer in Asian than in non‐Asian patients,^37^ although no notable difference in PFS was found between these subgroups in the ALTA trial in crizotinib‐refractory patients.^10^ The current study also assessed TTDR and found a reasonably high probability of continuation on the maximum/peak dose of brigatinib at both 1 and 2 years.

The study had several limitations, mostly associated with the nature of claims data, including the fact that the data are not from a medical perspective, there is generally a lack of outcomes data, and it is impossible to analyse non‐insured health benefits items.^38^, ^39^, ^40^ For example, it was only possible to obtain information on treatments that are claimed through the National Health Insurance Service by South Korean health insurance, and it was not possible to obtain medical records of treatment that is not reimbursed (such as in clinical trials or early access programmes, before drugs are approved for reimbursement). However, few patients had a gap of >180 days between claims for crizotinib and brigatinib, indicating that most of the study population could have received crizotinib and brigatinib under the conditions of reimbursement. Of the 8% of patients who had a gap of >180 days, some who had progression on first‐line crizotinib may not have been able to start brigatinib continuously due to limited accessibility; another possible reason for the treatment gap is that, as noted earlier, it is not possible to obtain data for non‐insured items (e.g. clinical trials, patient support programmes) from claims data, so, under national insurance, it is possible to receive brigatinib or other ALK TKIs via such programmes in‐between crizotinib and brigatinib treatment. An additional limitation is that brain metastases were defined using a diagnostic code (C793, secondary malignant neoplasm of brain and cerebral meninges) during the pre‐index period and, although magnetic resonance imaging (MRI) is the standard method for diagnosing brain metastases in Korea, claims data do not allow complete certainty that all brain metastases were diagnosed by MRI, whether brain metastases were symptomatic or what prior treatment, if any, patients received for brain metastases. Another limitation was that the specific cause of death could not be determined from HIRA data and, therefore, OS estimates were based on data that included non‐disease‐related deaths. Strengths of the study include the well‐defined patient population (patients moving from first‐line crizotinib to second‐line brigatinib) and the relatively long duration of follow‐up. In addition, the use of nationwide claims data means the results can be generalised to all patients using brigatinib as second‐line therapy for *ALK+* NSCLC in South Korea.

## 5 CONCLUSIONS

This is the first real‐world study of brigatinib treatment in a nationwide cohort of crizotinib‐refractory *ALK*+ NSCLC patients in South Korea. The study involved a large, well‐defined group of patients who received brigatinib as second‐line therapy. During a median 18 months of follow‐up, there was a high level of adherence to treatment, a longer treatment duration than reported for published clinical trials, and a favourable 2‐year OS rate. Overall, the results suggest that brigatinib is of benefit when administered as second‐line treatment in patients with crizotinib‐refractory *ALK+* NSCLC treated in a real‐world setting in South Korea.

## AUTHOR CONTRIBUTIONS

**Jeong Eun Lee:** Methodology (equal); writing – original draft (equal); writing – review and editing (equal). **Jin Hyun Nam:** Conceptualization (equal); data curation (equal); formal analysis (equal); methodology (equal); writing – review and editing (equal). **Sun Hong Kwon:** Conceptualization (equal); data curation (equal); formal analysis (equal); methodology (equal); writing – review and editing (equal). **Bo Kyung Kim:** Conceptualization (equal); project administration (equal); writing – review and editing (equal). **Seung Min Ha:** Conceptualization (equal); project administration (equal); supervision (equal); writing – review and editing (equal).

## FUNDING INFORMATION

The study was funded by Takeda Pharmaceuticals Korea Co., Ltd.

## CONFLICT OF INTEREST STATEMENT

J.E. Lee and J.H. Nam: none; S.H. Kwon: personal and institutional, research grant: Takeda; B.K. Kim and S.M. Ha: full or part‐time employment: Takeda.

## ETHICAL APPROVAL

The study was approved by the institutional review board of Sungkyunkwan University, Suwon, Republic of Korea. Informed consent was not required because the study used anonymised and de‐identified claims data.

## Data Availability

The datasets, including the redacted study protocol, redacted statistical analysis plan and individual participant data supporting the results reported in this article, will be made available within 3 months from the initial request to researchers who provide a methodologically sound proposal. Data will be provided after de‐identification, in compliance with applicable privacy laws, data protection and requirements for consent and anonymization.
